# Hospitals and the Spirit Tax

**Published:** 1915-07-17

**Authors:** 


### HOSPITALS AND THE SPIRIT TAX.

When, on the Committee stage of the Finance Bill, Mr. Bridgeman withdrew his clause providing for the use of duty-free spirit by hospitals, he intimated that it was his intention to bring forward his proposal on the Report stage. The Report stage was reached on Tuesday, but for good reasons, no doubt, Mr. Bridgeman did not carry out his expressed intention. It may be that on a future occasion there will be a more favourable opportunity of inserting the clause—or a similar one—in another Bill, but for the present, at any rate, the proposal appears to have been dropped. For this, which is nothing short of a tremendous disappointment for hospital authorities, thanks are mainly due to the British Medical Association, whose attitude throughout the whole unfortunate business has been extraordinary and apparently inexplicable.

The reasons for the Association's opposition, as put forward by its chief spokesman in the House of Commons, were trivial and unconvincing, and yet the House seems to have been impressed by them merely because they represented the views of an organisation which is supposed to stand for the best interests of the medical profession. A demand for a modification of the clause could have been understood; a reasoned amendment of the definition of a "public hospital" or a proposal to increase the safeguards could have been considered sympathetically, but it passes our comprehension to understand what was behind the point-blank refusal to accept the greatest gift that has ever been proffered to the sick poor in the history of the world. The opposition came as a surprise, but the defeat will not have been wholly in vain if the British Hospitals Association learns from it the importance of securing a spokesman in the House. The British Hospitals Association in the past has had to contend with a certain insularity, deliberately encouraged for understandable ends by many chairmen of the great institutions, among individual officers in high places who ought to be active supporters of it. The consequence has been that, at the critical moment, neither the hospitals as a whole nor the British Hospitals Association were able to bring united pressure to bear. The result has been temporary disaster.

The remedy is for the chairmen of the principal and of representative hospitals to join the Association and take a personal and active part in guiding its policy and proceedings. Further, the individual superintendents should spontaneously make it their business in self-protection to give time to the work of the Association. Once this necessary reorganisation, with its added strength, is supplied, the Association will secure that its collective voice shall be heard in Parliament, and that its work will be welcomed everywhere by public and hospital opinion.

It is roughly estimated that duty-free spirit would represent a saving to the hospitals of Great Britain of something in the region of £30,000 a year, which is equivalent to a legacy of three-quarters of a million sterling. What mandate had the B.M.A. from its constituents to reject this magnificent offer? The Association not only refused the bequest, but actually pleaded with members of Parliament not to grant it. "Representations have been made by our Association to the Chancellor of the Exchequer," wrote the Medical Secretary of the British Medical Association in a circular letter to members, "that the clause may be dropped, and that the whole question of duty-free alcohol in medicine be referred to a Special Committee for consideration and report. We earnestly ask your support in the House in support of this recommendation, as under the Finance Act the position of hospitals is not in any way worsened in regard to the use of spirits. Our Association are of opinion that the suggestion of delay is reasonable." Had it not been for this urgent plea there is no reason to suppose that the clause would not have been inserted in the Finance Bill, and the responsibility for its rejection rests mainly with the British Medical Association. One can perhaps understand the motives which prompted the chemists to oppose a measure which provided for a supply of cheap tinctures to hospitals, but the British Medical Association has no mandate to impoverish charitable institutions at a time when their resources have been so severely tested by the strain of the war. What will the hundreds of members at the Front have to say when on their return home they are told that their Association has strenuously resisted a concession so long fought for by the hospitals?

